# Energy Efficient Constellation for Wireless Connectivity of IoT Devices

**DOI:** 10.3390/s20143991

**Published:** 2020-07-17

**Authors:** Lorenzo Mucchi, Luca Simone Ronga, Sara Jayousi

**Affiliations:** 1 Department of Information Engineering, University of Florence, 50139 Florence, Italy; sara.jayousi@pin.unifi.it; 2 Leonardo SPA, Cyber-Security Division, Science and Technology Directorate, 50013 Campi Bisezio, Italy; luca.ronga@ieee.org

**Keywords:** energy efficient communication, MAP detector, zero energy symbol, signal constellation

## Abstract

Reducing energy consumption is one of the most important task of the approaching Internet of Things (IoT) paradigm. Existing communication standards, such as 3G/4G, use complex protocols (active mode, sleep modes) in order to address the waste of energy. These protocols are forced to transmit when one frame is only partially filled with information symbols. The hard task to adapt the power-saving mode with low latency to the discontinuity of the source is mainly due to the fact that the receiver cannot know a priori when the source has something to transmit. In this paper, we propose a modified signalling/constellation which can save energy by mapping a zero-energy symbol in the information source. This paper addresses the fundamentals of this new technique: the maximum a posteriori probability (MAP) criterion, the probability of error, the (energy) entropy, the (energy) capacity as well as the energy cost of the proposed technique are derived for the binary signalling case.

## 1. Introduction

Internet of things (IoT), mobile computing (MC), pervasive computing (PC), wireless sensor networks (WSNs), and, most recently, cyber-physical systems (CPS) are the promising research areas for the future smart world [1,2]. However, as technology and solutions progress in each of these fields, connectivity and energy consumption are always two of the most hard tasks to be handled [3,4,5,6,7].

Both the 5G and the next generation 6G wireless systems aim at dramatically increasing the throughput and latency performances, but also require to address low-power low-rates internet of things (IoT) devices with unseen energy efficiency targets. The desired efficiency requires substantial improvements at all layers. At the physical layer, orthogonal frequency division multiplexing (OFDM) modulation will be likely substituted by the promising filter-bank multi-carrier [8], which breaks the constraints imposed by DFT on the carrier modulating pulse shape. Signal constellation is also a potential field of improvements. As IoT concept spreads, the operational situations where sporadic transmission of burst data become more frequent. Efficient ways to exploit the discontinuity of radio sources may be of interest for several factors, ranging from energy efficiency to reduced radio interference. This work proposes a physical layer approach to burst transmissions management, where the signalling overhead is replaced by a contained increment of error ratio. This tradeoff is analysed in the paper, identifying the operational contexts where the proposed solution is convenient.

### 1.1. Literature Review

In the literature, there are various methods to exploit the discontinuity of the information sources to save energy. The LTE architecture exploits the idea of Discontinuous Reception (DRX) and Discontinuous Transmission (DTX) to provide a concrete solution to the power saving. LTE provides methods for the user equipment (UE) to micro-sleep even in the active state to reduce power consumption while providing high QoS and connectivity [9,10]. If no transmissions are detected for a specific period, the UE is allowed not to monitor the downlink control channel in the given subframe and to go to power saving mode for a short period (short DRX cycle). This is a simple method to keep the UE alive, especially useful for bursty traffic, as opposed to regular. If for several DRX Short Cycles there are no transmissions, a long DRX Cycle is activated. The long DRX cycle is used during UE’s inactivity periods, when it only has to check the control channels and no resources are assigned. During both short and long DRX Cycles, the radio-frequency (RF) modem is turned on periodically for several consecutive subframes to listen to the control channels. When data activity is detected both in downlink or uplink, the short DRX cycle for UE is triggered, increasing the responsiveness and connectivity of UE. Basically, the LTE standard, and others similarly, exploit the silence of the source using two power save periods with different granularity to adapt to different discontinuous sources. During these power saving periods, the RF module is activated regularly to control if transmissions are planned, thus consuming energy as well. Moreover, only two cycles (short and long) seem to be less adaptive to the traffic generated by discontinuous sources.

More recently, several papers have been published on this topic (see [11,12,13] and references therein). In particular, in [14] a joint optimization of constellation size, energy allocation per bit and relay location assuming fixed end-to-end distance is proposed. In [15], an energy-efficient collaborative space–time block code (STBC) is proposed. In [16], a multi-dimensional compact constellations that minimize the average symbol energy is given, while in [17] the energy-saving gained from optimizing the constellation size is investigated. In [18], a multidimensional secure constellation (MSC) design to improve the energy efficiency, security, and bit error rate (BER) performance is described. In [19], an energy-based pulse amplitude modulation (PAM) constellation design framework for noncoherent detection in massive single-input multiple-output (SIMO) systems is proposed, while in [20] an energy-efficient multi-layer modulation constellation to manage the multiuser interference for broadcast system over Poisson channel is given. In [21], a symbol-level precoding for multiuser multiple-input single-output (MISO) to minimize the peak transmission energy over symbol time slots in downlink scenario is described. In [22], performance of a device-to-device (D2D) multiple-input and multiple-output (MIMO) communications using an amplify-and-forward relaying for various energy-efficient modulation schemes is investigated. In [23], a quadrature space-frequency index modulation (QSF-IM) scheme for 5G system energy-efficiency is proposed, while in [24] implementation scenarios of spatial modulation (SM) techniques for spectral and energy efficiency of 5G wireless networks are discussed. In [25], an energy-efficient framework for adaptively controlling the transmit power and data rate of the IoT device in order to minimize the energy-consumption in 5G networks is discussed, while in [26] and [27] index modulation is proposed as a key technique to provide spectral and energy efficiency in 5G networks. In [28], an orbital angular momentum (OAM) based, energy-efficient multidimensional coded modulation is proposed. In [29], an energy model for a cooperative amplify-and-forward (AF) relay system is proposed.

### 1.2. Our Contribution

Differently, in this paper, the authors propose an alternative signal constellation for data carriers in 5G/6G systems. The proposed constellation is characterized by the insertion of a zero-energy symbol that sources can use in the presence of variable information rate and discontinuous activity. The impact of this new signaling scheme is twofold. First data carriers modulated with the “zero” symbol to save energy. In presence of different traffic flows transported by the same bearer, the suppression of the entire frame is possible only when all flows are silent. The proposed scheme map silences with much more flexibility within the single frame producing a fine granularity in energy consumption. Low-rate sources woken up by events (i.e., sensors), face a high signaling latency unless athey re assigned dedicated continuous resources. The presence of a silent symbol in the constellation avoids the high layer activity signaling, thus dramatically reducing the service latency for impulsive low rate sources.

In this paper, we provide the baseline of the proposed energy efficient constellation, where a zero-energy symbol is added to the classical binary phase shift keying (BPSK). This constellation maps silence states of the information source to that symbol which corresponds to a zero energy signal transmitted from the antenna. As shown below, the presence of a zero energy symbol increases the energy efficiency for low activity sources and provides an instant resume when the source leaves the silence state. The proposed signaling scheme differs from OOK (on–off keying) for the presence of a complete energy balanced set of information signals. The concept can be extended to multilevel constellation (QAM) and the transmitted signal is physically (but not semantically) backward compatible. In other words, the zero-energy symbol is one legitimate symbol of the constellation that the receiver could expect to receive from the source. This fact reaches a two-fold benefit: a) there is no need to send additional control bits to inform the receiver that a silent state is active; b) there is no need to implement a wake up of the receiver, since the zero-symbol is directly detected by the demodulator. A sleep state of the receiver can be implemented anyway to save energy in case of very long periods of inactivity. In this case, the wake-up strategy is exactly the same of any receiver, according to the protocol implemented, e.g., wake up is bond to the reaching of a specific date and time, etc.

Section 2 provides the maximum-a-posteriori and maximum-a-priori criterion for the proposed signaling scheme for AWGN channel. In Section 3 some performance metrics are proposed for a fair comparison with the traditional constellations. Section 4 provides some commented numerical results while Section 5 provides an evaluation of the attained energy efficiency and channel capacity. In Section 6 there is a evaluation of the equivalent signaling overhead in traditional constellations. Section 7 has some concluding remarks.

## 2. MAP Criterion for Z-BPSK in AWGN Channel

The recall of the maximum a posteriori criterion (MAP) is reported in Appendix A. Since this paper aims to describe the fundamentals of the new proposed mapping technique, we focus hereafter on a simple binary antipodal constellation (BPSK). Extension to multilevel constellations (QAM) is straightforward and can be easily derived from the following results.

In the case of a binary signaling plus the zero symbol, the matched filter vector representation is scalar and the signal space is $s_{m}\in \left\{0,\sqrt{E_{1}},-\sqrt{E_{1}}\right\}$. It should be noted that $E_{1}$ does not equal the average signal energy $E_{s}$, which is

(1) $$E_{s}=E\left[s_{m}^{*}s_{m}\right]=\left(1-P_{0}\right)E_{1}$$

where $E_{1}$ is the energy per non-zero symbol. The received signal in the matched filter scalar representation is

(2) $$r=s_{m}+z$$

where *z* is Gaussian i.i.d. process with zero mean and variance $\sigma_{n}^{2}$. Under these conditions we have:

(3) $$\begin{array}{cc} p(r|s_{0})= & \frac{1}{\sqrt{2\pi }\sigma_{n}}e^{-\frac{r^{2}}{2\sigma_{n}^{2}}} \end{array}$$

(4) $$\begin{array}{cc} p(r|s_{1})= & \frac{1}{\sqrt{2\pi }\sigma_{n}}e^{-\frac{{\left(r-\sqrt{E_{1}}\right)}^{2}}{2\sigma_{n}^{2}}} \end{array}$$

(5) $$\begin{array}{cc} p(r|s_{2})= & \frac{1}{\sqrt{2\pi }\sigma_{n}}e^{-\frac{{\left(r+\sqrt{E_{1}}\right)}^{2}}{2\sigma_{n}^{2}}} \end{array}$$

If we assume the information symbols equally probable, we have from Equation (A5) in Appendix A

(6) $$\left\{\begin{array}{cc} P(s_{0})= & P_{0}\\ P(s_{1})= & P\left(s_{2}\right)=\frac{1-P_{0}}{2} \end{array}\right.$$

The MAP criterion applied to this case yields a decision for the symbols $s_{0}$ when

(7) $$p\left(r|s_{0}\right)P_{0}>p\left(r|s_{i}\right)\frac{1-P_{0}}{2}\;\forall i\neq 0.$$

By substituting Equation (3) in Equation (7) and taking the base-2 logarithm of both sides, we obtain the MAP decision region of symbol $s_{0}$ as

(8) $$\left\{\begin{array}{cc} r< & \frac{\sqrt{E_{1}}}{2}+\frac{\sigma_{n}^{2}}{\sqrt{E_{1}}}\ln\left(\frac{2P_{0}}{1-P_{0}}\right)=\theta_{0,1}\\ r> & -\frac{\sqrt{E_{1}}}{2}-\frac{\sigma_{n}^{2}}{\sqrt{E_{1}}}\ln\left(\frac{2P_{0}}{1-P_{0}}\right)=\theta_{-1,0} \end{array}\right.$$

i.e., $\theta_{0,1}$ and $\theta_{-1,0}$ are the boundary points between the decision region of symbol zero and symbol +1 and -1, respectively. In the special case when all symbols, including the zero-energy one, are equally probable, Equation (8) becomes

(9) $$-\frac{\sqrt{E_{1}}}{2}<r<\frac{\sqrt{E_{1}}}{2}$$

Analogously by defining the Euclidean distance $d_{m}$ of received signal from transmitted signal ${\bar{s}}_{m}$:

(10) $$d_{m}=d_{m}\left(\bar{r}\right)=\sqrt{\bar{r}-{\bar{s}}_{m}}$$

substituting into Equation (7) yields the decision region of symbol $s_{0}$ expressed as:

(11) $$d_{0}^{2}<d_{i}^{2}+2\sigma_{n}^{2}\ln\left(\frac{P(s_{0})}{P(s_{i})}\right)\;\forall i\neq 0$$

## 3. Probability of Error

In this section, the probability of error as a performance metric is derived and discussed. The probability of error is not the only metric addressed here, anyway. Energy entropy, energy capacity and energy cost are also evaluated in the following sections.

Due to the presence of the zero-energy symbol in the constellation, the possible errors committed by the receivers are more complex than the traditional case.

Table 1 shows the possible types of mistakes that a receiver can commit. These classes of errors, false activity, false zero and symbol error produce three types of corresponding error probabilities: $P_{FA}$, $P_{FZ}$ and $P_{SE}$. We also define the aggregate probability of error as

(12) $$P_{E}=P_{FA}+P_{FZ}+P_{SE}$$

In the Z-BPSK case, considering the decision region boundaries from Equation (8) we can write

(13) $$P_{FA}=P_{FA}|s_{0}=P\left(s_{0}\right)\left(1-\int_{-\theta_{0,1}}^{\theta_{0,1}}p\left(r|s_{0}\right)dr\right)$$

where $\theta_{0,1}$ is the boundary point between the decision region of symbol zero and symbol +1. From Equation (8) we can observe that the boundary between symbol −1 and symbol zero is $\theta_{-1,0}=-\theta_{0,1}$.

For what concern the probability of false zero we can write

(14) $$P_{FZ}=P_{FZ}\left|s_{1}+P_{FZ}\right|s_{2}=P\left(s_{1}\right)\int_{-\theta_{0,1}}^{\theta_{0,1}}p\left(r|s_{1}\right)dr+P\left(s_{2}\right)\int_{-\theta_{0,1}}^{\theta_{0,1}}p\left(r|s_{2}\right)dr$$

While the probability of symbol error is

(15) $$P_{SE}=P_{SE}\left|s_{1}+P_{SE}\right|s_{2}=P\left(s_{1}\right)\int_{-\infty }^{-\theta_{0,1}}p\left(r|s_{1}\right)dr+P\left(s_{2}\right)\int_{\theta_{0,1}}^{+\infty }p\left(r|s_{2}\right)dr$$

In the case of AWGN channel the above equation can be expressed by using the *Q*-funtion

(16) $$Q\left(x\right)=\frac{1}{\sqrt{2\pi }}\int_{x}^{+\infty }e^{-\frac{x^{2}}{2}}dx,\;x\geq 0$$

resulting in

(17) $$\begin{array}{cc} P_{FA}= & 2P_{0}Q\left(\frac{\theta_{0,1}}{\sigma_{n}}\right) \end{array}$$

(18) $$\begin{array}{cc} P_{FZ}= & \left(1-P_{0}\right)\left[Q\left(\frac{\sqrt{E_{1}}-\theta_{0,1}}{\sigma_{n}}\right)-Q\left(\frac{\sqrt{E_{1}}+\theta_{0,1}}{\sigma_{n}}\right)\right] \end{array}$$

(19) $$\begin{array}{cc} P_{SE}= & \left(1-P_{0}\right)Q\left(\frac{\sqrt{E_{1}}+\theta_{0,1}}{\sigma_{n}}\right). \end{array}$$

The aggregate probability of error defined in Equation (12) results

(20) $$P_{E}=2P_{0}Q\left(\frac{\theta_{0,1}}{\sigma_{n}}\right)+\left(1-P_{0}\right)Q\left(\frac{\sqrt{E_{1}}-\theta_{0,1}}{\sigma_{n}}\right)$$

## 4. Numerical Analysis and Comments

The error probabilities computed in Section 3 can be compared to the traditional BPSK signaling. For a fair comparison we set the constant symbol energy of BPSK equal to the averaged symbol energy in Z-BPSK defined in Equation (1), i.e., $E_{s}^{\left[BPSK\right]}=E_{s}^{\left[Z-BPSK\right]}=\left(1-P_{0}\right)E_{1}$. This means that the probability of error of Z-BPSK and BPSK are computed with the same SNR $\gamma =\frac{E_{s}}{N_{0}}$.

**Figure 1:**
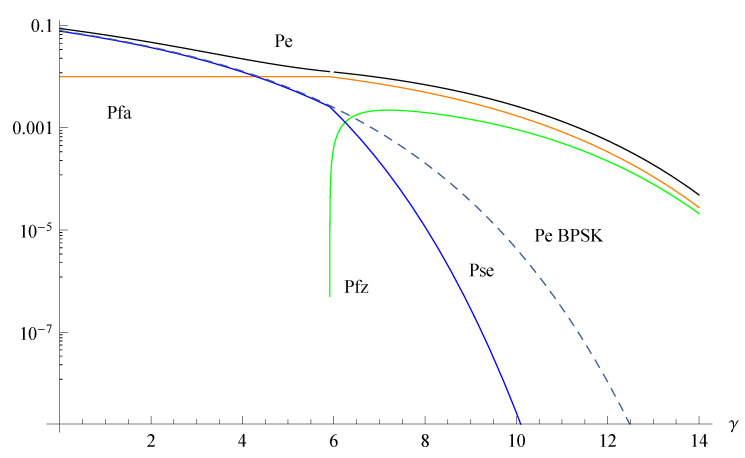
Error probabilities of the Z-binary phase shift keying (BPSK) and BPSK for $P_{0}=0.01$ as a function of the SNR $\gamma =E_{s}/N_{0}$.

**Figure 2:**
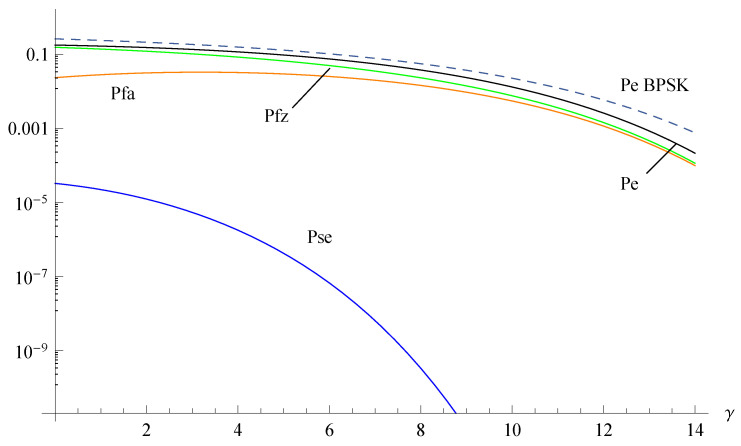
Error probabilities of the Z-BPSK and BPSK for $P_{0}=0.8$ as a function of the SNR $\gamma =E_{s}/N_{0}$.

Figure 1 and Figure 2 show the three error components $P_{FA}$, $P_{FZ}$, $P_{SE}$ separately, the compound sum $P_{E}$ for the proposed Z-BPSK signaling as well as the symbol error probability for the legacy BPSK. The graph reports the probabilities versus the signal-to-noise ratio $\gamma =E_{s}/N_{0}$, where $N_{0}=2\sigma_{n}^{2}$ is the noise spectral density, for two values of $P_{0}=\left\{0.01,0.8\right\}$. These two values have been selected to represent two extreme situations: when the probability of non-activity (silence) is very low (0.01), and when the probability of non-activity is high (0.8). When the probability of the zero energy symbol is low, the performance present two different behaviors: at low SNRs the zero energy decision region collapses, $P_{FA}$ is constantly equal to $P_{0}$, $P_{FZ}$ drops to zero and the symbol error probability $P_{SE}$ approaches $P_{E}$ of legacy BPSK. For high SNRs, the zero symbol is progressively detected and the $P_{SE}$ outperforms the legacy BPSK.

A completely different situation is found in Figure 2. For a high probability $P_{0}$, the zero symbol detection errors still dominate the performance but the constellation performs globally better than traditional BPSK.

The above comparisons do not take into account the different amount of information the signals are carrying. In a single symbol, Z-BPSK can expose three different states that legacy BPSK cannot deliver. For a more fair comparison, an additional correction has to be made. Let us consider a frame with $N_{f}$ three-state symbols. If the probability of emission of a zero symbol is $P_{0}<1/N_{f}$ we can assume optimistically that we will have at most a single zero-energy symbol in the frame. For a legacy BPSK we need to send additional symbols in order to map the eventual occurrence (and position) of the zero energy symbol in the frame. These overhead symbols are $N_{s}=\log_{2}\left(N_{f}+1\right)$. Hence, with a legacy BPSK, we should transmit $N_{f}+N_{s}$ symbols to deliver $N_{f}$ three-state symbols with a given $P_{0}$. These additional symbols are transmitted at the expense of more energy. Therefore, a fair comparison would mean to set:

(21) $$E_{s}^{\left[BPSK\right]}=E_{s}^{\left[Z-BPSK\right]}\frac{N_{f}}{N_{f}+N_{s}}$$

where $E_{s}^{\left[BPSK\right]}$ is the energy per symbol of BPSK and $E_{s}^{\left[Z-BPSK\right]}$ is the energy per symbol of the Z-BPSK. Note that the above computation is biased in favor of BPSK since if there are more than one zero energy symbol in a frame, the number of additional (overhead) symbols is higher.

## 5. Energy Entropy and Energy Capacity

In this section, we show how the proposed signal constellation, i.e., zero energy symbol mapping, can reach benefits from the energy consumption point of view. To show this we have derived the source entropy and the channel capacity of the Z-BPSK and compared it with the BPSK known results.

### 5.1. Entropy

Given a BPSK signalling, the entropy of the binary source $H_{B}\left(X\right)$ is well known [30]

(22) $$H_{B}\left(X\right)=p\left(s_{1}\right)\log p\left(s_{1}\right)+p\left(s_{2}\right)\log p\left(s_{2}\right)$$

where *X* is a discrete random variable with possible values $\left\{s_{1},s_{2}\right\}$. The values $s_{1}$ and $s_{2}$ can be interpreted as the symbols emitted by the informative source.

In the proposed Z-BPSK, the zero symbol $s_{0}$ has to be added to the binary symbols $s_{1}$ and $s_{2}$. Thus, the entropy of the Z-BPSK $H_{z}\left(X\right)$ can be defined as

(23) $$H_{Z}\left(X\right)=-P_{0}\log P_{0}-\left(1-P_{0}\right)\log\frac{1-P_{0}}{2}$$

where $P_{0}=P\left(s_{0}\right)$ is the probability of generation of the zero symbol. The probability of emitting a non-zero symbol is supposed equiprobable $p\left(s_{1}\right)=p\left(s_{2}\right)=\frac{1-P_{0}}{2}$.

Defining the energy entropy as the number of symbols that a source can emit per energy unit

(24) $$H_{1}\left(X\right)≜\frac{H(X)}{E_{s}},$$

we can compare the bit-per-joule curves for the Z-BPSK and the BPSK. The energy per symbol $E_{s}$ is different for the Z-BPSK and the BPSK (Equation (1))

(25) $$\begin{array}{ccc} E_{s}^{\left[BPSK\right]} & = & E_{1} \end{array}$$

(26) $$\begin{array}{ccc} E_{s}^{\left[Z-BPSK\right]} & = & \left(1-P_{0}\right)E_{1} \end{array}$$

**Figure 3:**
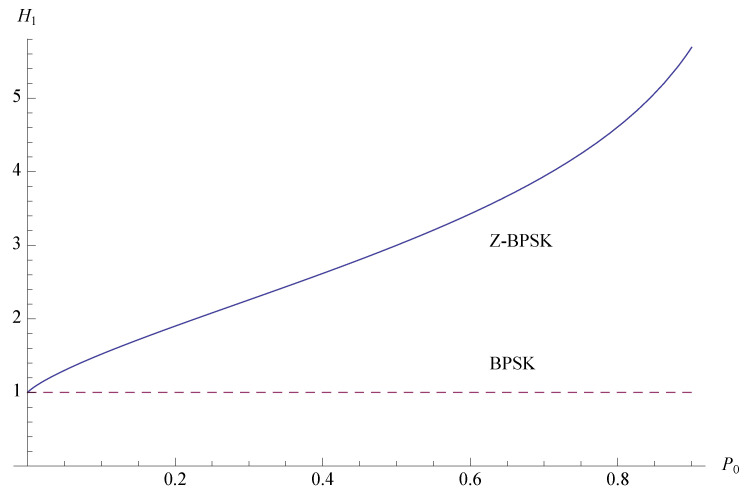
Entropy per unit of energy of the Z-BPSK (solid line) and classic BPSK (dashed line) as a function of the probability of emission of the zero symbol.

The result of the comparison is shown in Figure 3. If the probability of emission of the zero symbol increases, the entropy of the Z-BPSK grows significantly over the entropy of the BPSK.

### 5.2. Capacity

The channel capacity $C_{B}$ of a binary erasure channel (BEC) is well known [30]

(27) $$C_{B}=1-P_{SE}$$

where $P_{SE}$ is the symbol error probability.

The transition probabilities $P_{SE},P_{FA},P_{FZ}$ in Equation (17) and the probability of zero symbol emission $P_{0}$ can be used to derive the capacity $C_{Z}$ for the Z-BPSK

(28) $$C_{Z}=\sum_{i=0}^{2}\sum_{j=0}^{2}p\left(x_{j}\right)p\left(y_{i}|x_{j}\right)\log\frac{p(y_{i}|x_{j})}{p(y_{i})}$$

where $x_{j}$ is the symbol emitted by the source($\left\{s_{0}=0,s_{1}=1,s_{2}=-1\right\}$ in case of Z-BPSK) and $y_{i}$ is the symbol received by the destination. After some operations, the capacity can be written as

(29) $$\begin{array}{c} C_{Z}=P_{FZ}\log\left(\frac{P_{FZ}}{P_{FZ}-3P_{FZ}P_{0}+P_{0}}\right)+P_{SE}\left(1-P_{0}\right)\log\left(\frac{P_{SE}}{P_{FA}P_{0}+\left(1-P_{FA}\right)\left(\frac{1-P_{0}}{2}\right)}\right)+\\ +\left(1-P_{FZ}-P_{SE}\right)\left(1-P_{0}\right)\log\left(\frac{1-P_{FZ}-P_{SE}}{P_{FA}P_{0}+\left(1-P_{FA}\right)\left(\frac{1-P_{0}}{2}\right)}\right)+\\ +2P_{0}P_{FA}\log\left(\frac{P_{FA}}{P_{FA}P_{0}+\left(1-P_{FA}\right)\left(\frac{1-P_{0}}{2}\right)}\right)+P_{0}\left(1-2P_{FZ}\right)\log\left(\frac{1-2P_{FZ}}{P_{FZ}-3P_{FZ}P_{0}+P_{0}}\right) \end{array}$$

If the transitions between source and destination have no error, the capacity reaches asymptotically the source entropy, as the classical BPSK

(30) $$\lim_{P_{SE}\to 0}C_{B}=1$$

(31) $$\lim_{P_{SE},P_{FA},P_{FZ}\to 0}C_{Z}=-P_{0}\log P_{0}-\left(1-P_{0}\right)\log\left(\frac{1-P_{0}}{2}\right)$$

It is important to note that, unlike $C_{B}$, the capacity of the Z-BPSK is greater than one for $0<P_{0}<0.55$. In fact,

(32) $$\begin{array}{ccc} \lim_{P_{SE},P_{FA},P_{FZ},P_{0}\to 0}C_{Z} & = & 1 \end{array}$$

(33) $$\begin{array}{ccc} \lim_{P_{SE},P_{FA},P_{FZ}\to 0,P_{0}\to 1}C_{Z} & = & 0 \end{array}$$

The capacity of the proposed constellation, i.e., the insertion of a zero-energy symbol, reaches a greater capacity compared to classic BPSK, in error-free channels.

The energy capacity, as defined in [31], can be thus derived

(34) $$C_{1}≜\frac{C}{E_{S}}.$$

**Figure 4:**
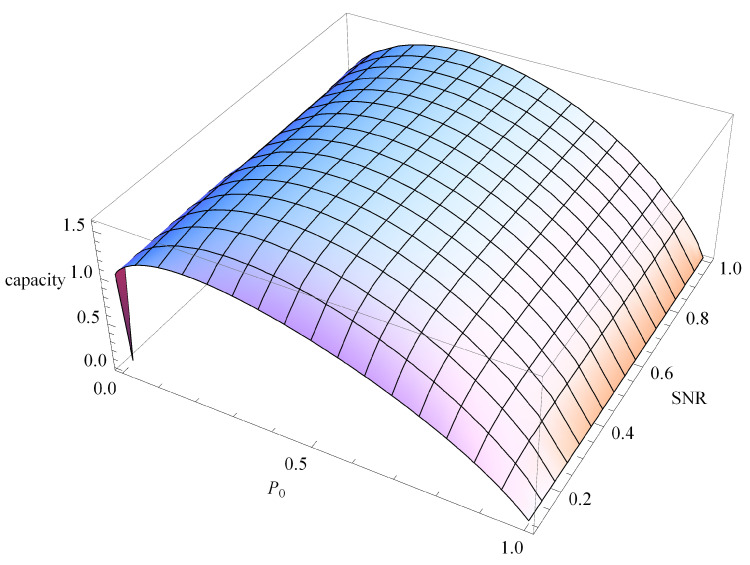
Capacity of the Z-BPSK as a function the probability of emission zero symbol and SNR.

We can then compare the bit-per-joule curves for the capacity of the Z-BPSK and the BPSK. The energy per symbol $E_{s}$ is different for the Z-BPSK and the BPSK, as reported in Equation (25). The capacity of Z-BPSK ($C_{Z}$) is shown in Figure 4 as a function of the probability of emission of zero symbol ($P_{0}$) and SNR.

**Figure 5:**
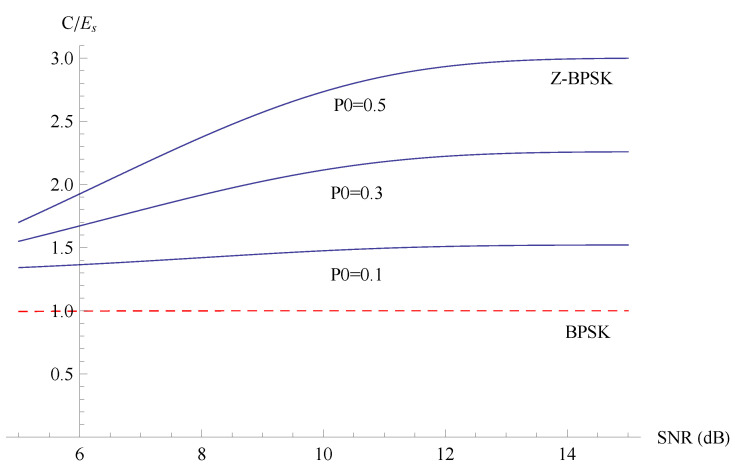
Energy capacity of the Z-BPSK and BPSK as a function of the SNR for different values of $P_{0}$.

**Figure 6:**
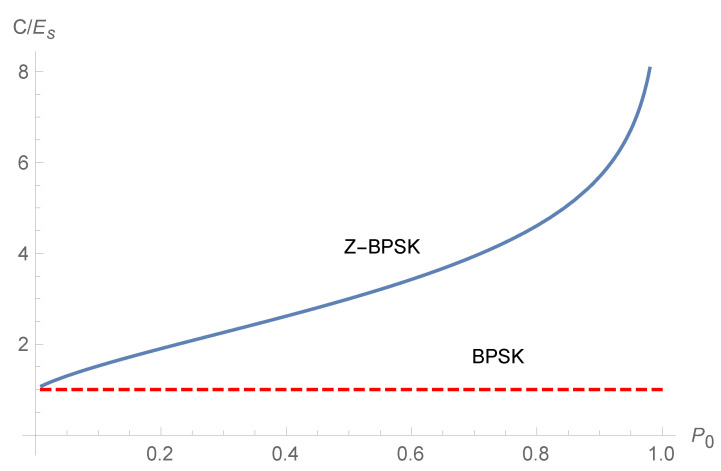
Energy capacity of the Z-BPSK and BPSK as a function of $P_{0}$.

The energy capacity of the Z-BPSK and BPSK are compared in Figure 5 as a function of the SNR for different values of $P_{0}$, while in Figure 6 the energy capacity is drawn as a function of $P_{0}$ for a fixed SNR. The SNR ($E_{s}/N_{0}$) in Figure 5 is calculated by fixing the energy per symbol $E_{s}=1$ and varying the noise power $N_{0}$.

The results clearly show how the proposed zero-energy mapping can assure a higher energy efficiency, in terms of higher bit-per-joule, even for low probability of emission of zero symbol.

## 6. Energy Cost

Most existing wireless mobile networks (including 3G and LTE) employ Discontinuous Reception (DRX) to conserve the power of mobile user devices (MSs). DRX allows an idle MS to power off the radio transceiver for a predefined period (called the DRX cycle) instead of continuously listening to the radio channel. The main idea is to let the MS switch off the radio receiver for several frames if there are not bits to be transmitted. While in sleep mode, the MS listens at the control channel in order to wake up (active mode) if the base station (BS) indicates that there are frames to be transmitted. All these BS-MS control bits (which are not information) are sent by means of additional power. Furthermore, one frame which is partially filled with non-zero symbols has to be transmitted anyway, consuming energy for nothing. Or, the frame is buffered waiting to accumulate non-zero symbols to fill the frame, but this policy does not apply well to delay-sensitive services such as voice and web browsing, or when there are several information sources with constant but different symbol emission rates.

**Figure 7:**
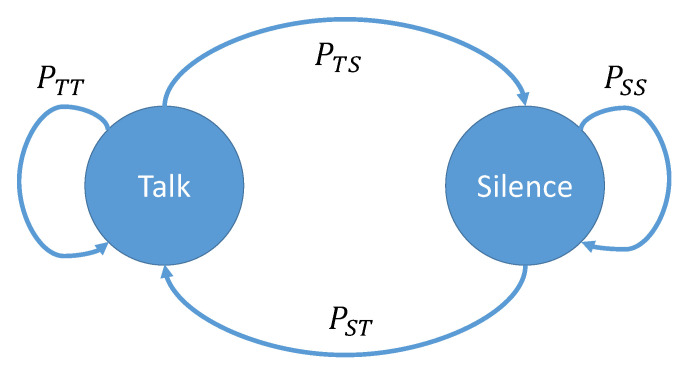
Markov chain modelling the source emission of zero and non-zero symbols.

Let us focus on a information source and model it with a two-state Markov chain: Talk State (T) and Silence State (S), as depicted in Figure 7.

Given the transition probabilities $P_{TS}$, from Talk to Silence, and $P_{ST}$ from Silence to Talk, the probability of Talk $P_{T}$ and Silence $P_{S}$ can be derived

(35) $$\begin{array}{ccc} P_{T} & = & \frac{P_{ST}}{P_{ST}+P_{TS}} \end{array}$$

(36) $$\begin{array}{ccc} P_{S} & = & 1-P_{T}=\frac{P_{TS}}{P_{ST}+P_{TS}}=P_{0} \end{array}$$

The probability $P_{S}$ is equivalent to the probability of zero-symbol emission that we depicted with $P_{0}$ in the previous sections. As known, the probability to stay in a state and to leave a state sum up to 1, e.g., $P_{TT}+P_{TS}=1$.

Let us focus now on a traditional frame-based transmission. In order to derive the average energy per transmitted frame ($E_{f}$), we have to first calculate the probability of remaining in the silence state for a number of symbols (*n*) to fill entirely one frame ($N_{f}$)

(37) $$P\left\{n=N_{f}\right\}={\left(1-P_{ST}\right)}^{N_{f}-1}P_{ST}\;$$

since in a traditional BPSK transmission, if at least one symbol is emitted in a frame, the frame has to be transmitted entirely, using an energy equal to $N_{f}E_{1}$, where $E_{1}$ is the energy per non-zero symbol. If the system remains in the silence state for an interval equal to $N_{f}$ symbols, then that frame is not transmitted and the energy cost is zero, for that frame. Thus, during a silence state, the average energy per frame can be written as

(38) $$E_{fs}=\sum_{i=1}^{N_{f}-1}P_{ST}{\left(1-P_{ST}\right)}^{N_{f}-1}N_{f}E_{1}=N_{f}E_{1}\left[1-{\left(1-P_{ST}\right)}^{N_{f}-1}\right]$$

On the contrary, during a talk state, the average energy per frame is

(39) $$E_{ft}=P_{T}N_{f}E_{1}$$

Thus, the overall average energy per frame of the BPSK is

(40) $$E_{f}^{\left[BPSK\right]}=E_{fs}+E_{ft}=N_{f}E_{1}\left(P_{T}+\left(1-{\left(1-P_{ST}\right)}^{N_{f}-1}\right)\right)$$

Let us now consider the Z-BPSK scheme. In this case the silent state is directly mapped into the symbol constellation (through a symbol “0”), and every time the source is in a silent state it simply transmits a zero-energy symbol. Thus, in this case, the average energy per frame can be written as

(41) $$E_{f}^{\left[Z-BPSK\right]}=N_{f}E_{1}\left(1-P_{0}\right)=N_{f}E_{1}\frac{P_{ST}}{P_{ST}+P_{TS}}$$

**Figure 8:**
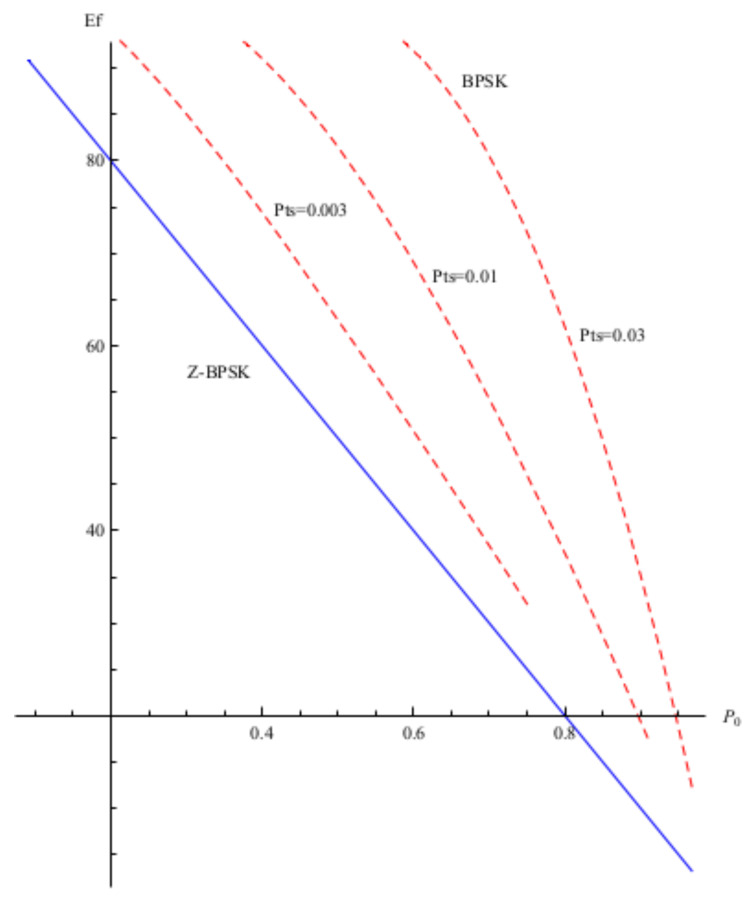
Average energy per frame $E_{f}$ of the Z-BPSK (solid line) and BPSK (dashed line) as a function of the probability of zero symbol emission $P_{0}$ for different values of the probability of transition from talk to silence state $P_{TS}$.

The average energy-per-frame of a classical signalling method (such as a frame-based BPSK) and the proposed zero-energy mapping method (Z-BPSK) can be compared in order to highlight the cost in terms of energy to transmit a frame of symbols, as a function of the transition probabilities between the talk and silence states. The results are shown in Figure 8. The proposed method is able to reduce the cost of the energy by a factor 20 and more, depending on how frequent the zero symbol emission is and, in particular, the transition from talk to silence. For example, with $P_{0}=0.4$, which is typical, e.g., of voice services, and $P_{TS}=0.003$, the proposed method compared to the classical one was able to save 34% of energy, while with $P_{TS}=0.01$ the energy saved growed up to 84%. A low value of $P_{TS}$ means that the transition between the talk and silence state has low probability to occur. A more variable source, i.e., frequent zeros distributed in the stream like several aggregated sources with different constant rate, leads to more frequent transition between talk and silence state and a consequent growing of the energy saved with our method.

## 7. Conclusions

In this paper, a modification of the alphabet of the symbols transmitted by the source is proposed. In particular, a zero-energy symbol is mapped directly into the constellation. This makes the zero symbol part of the information sent to the receiver, without any need for additional control bits or strategies. The map criterion, the probability of error, the entropy and the capacity of the proposed zero-energy mapping are derived for binary signalling case (Z-BPSK) and compared to the classic binary signalling (BPSK). The results show that the proposed concept performs as the traditional modulation, while saving energy, in particular when the probability of zero-symbol emission is significant, i.e., when the source experiences many silent periods.

The proposed solution would be useful to save energy and overhead in all the contexts where bursty traffic of data is produced, like in voice or browsing services. The results show that our solution is able to save significant amount of energy with the same probability of error of a traditional system in the case of bursty traffic.

## Figures and Tables

**Table 1:** Error types.

Sent	Detected	Error
$s_{0}$	$s_{0}$	no error
$s_{0}$	$s_{1}$	false activity
$s_{0}$	$s_{2}$	false activity
$s_{1}$	$s_{0}$	false zero
$s_{1}$	$s_{1}$	no error
$s_{1}$	$s_{2}$	symbol error
$s_{2}$	$s_{0}$	false zero
$s_{2}$	$s_{1}$	symbol error
$s_{2}$	$s_{2}$	no error

## Appendix A. Maximum A-Posteriori Criterion

We first recall the base equations for maximum a-posteriori (MAP) criterion. Let us denote with P(·) the discrete probability function and with p(·) the probability density function (pdf). Given the a-posteriori observation of received vector $\bar{r}$, we define the a-posteriori probability of signal ${\bar{s}}_{m}$ as [32]

(A1) $$P({\bar{s}}_{m}|\bar{r})\;m=0...M-1$$

where *M* is the number of symbols in the constellation. By using the Bayes’s rule [32]:

(A2) $$P\left({\bar{s}}_{m}|\bar{r}\right)=\frac{p\left(\bar{r}|{\bar{s}}_{m}\right)P\left({\bar{s}}_{m}\right)}{p(\bar{r})}$$

we obtain that the a-posteriori probability is function of a-priori symbol probability and conditional pdf of the received vector observed. At denominator of Equation (A2) we find the term $p(\bar{r})$ which can be written as

(A3) $$p\left(\bar{r}\right)=\sum_{m=0}^{M-1}P\left({\bar{s}}_{m}\right)p\left(\bar{r}|{\bar{s}}_{m}\right)$$

For an information source which maps also the zero symbol we can write

(A4) $$P\left({\bar{s}}_{m}\right)\left\{\begin{array}{cc} P_{0} & m=0\\ P_{m} & m=1\cdots M-1 \end{array}\right.$$

where $P_{0}$ is the probability of emission of a zero-energy symbol and $P_{m}$ is the probability of emission of the *m*-th non-zero symbol. When the non-zero energy information symbols are equally probable, Equation (A4) can be rewritten as

(A5) $$P\left({\bar{s}}_{m}\right)\left\{\begin{array}{cc} P_{0} & m=0\\ \frac{1-P_{0}}{M-1} & \mathrm{otherwise} \end{array}\right.$$

The MAP criterion is adopted when the decoded symbol which maximizes Equation (A2) is selected. Thus, the detected index $\hat{m}$ is

(A6) $$\hat{m}=\arg\max_{m}P\left({\bar{s}}_{m}|\bar{r}\right)$$

Since denominator of Equation (A2) is independent of *m*, MAP criterion can be written as

(A7) $$\hat{m}=\arg\max_{m}p\left(\bar{r}|{\bar{s}}_{m}\right)P\left({\bar{s}}_{m}\right)$$
